# Service provision for Frailty in European Emergency Departments (FEED): a survey of operational characteristics

**DOI:** 10.1186/s13049-024-01234-w

**Published:** 2024-07-29

**Authors:** Christophe A. Fehlmann, Kara Mc Loughlin, Emma Jane Cosgriff, John Francis Ferrick, James David van Oppen, European Taskforce for Geriatric Emergency Medicine, Kara Mc Loughlin, James David van Oppen, Timothy Coats, Simon Conroy, Bas de Groot, Pieter Heeren, Stephen Lim, Jacinta Lucke, Simon Mooijaart, Christian H. Nickel, Rose Penfold, Katrin Singler, Pieter Heeren, Françoise Steenebruggen, Valerie Sterckx, Ivan Brdar, Pavla Libicherová, Frédéric Balen, Céline Bianco, Xavier Dubucs, Jérémy Guenezan, Stefanie Apfelbacher, Othon Fraidakis, Varvara Fyntanidou, Szabolcs Gaál, Anna Björg Jónsdóttir, Maria Cremin, Mary Kelly, Claire McAteer, Elizabeth Moloney, Ciara Sankey, Lisa Sibthorpe, Maria Beatrice Zazzara, Rene Alexander Camilleri, Paul Zammit, Sophie M. Coffeng, Jacinta Lucke, Rosalinde Smits, Miguel Alberto Rizzi Bordigoni, Santiago Castejón-Hernández, Lupe del Rocio Coronel Chumbi, Sira Aguiló Mir, Eduardo Enrique Padilla, Wojciech Rojewski-Rojas, Davide Fadini, Natalie Sabrina Jegerlehner, Christian H. Nickel, Enrico Zucconi, Hüseyin Avni Demir, Zerrin Defne Dundar, Ramazan Güven, Mehmet Akif Karamercan, Fulya Kose, Özgür Söğüt, Ismail Tayfur, Lucy Abbott, James Adams, Janice Bernardo, Leanne Brown, Joel Burton, Renate Claassen, Jamie Cooper, Ruth Heyes, Calvin Lightbody, Jane Masoli, David Mawhinney, Stephen McKenzie, Nicola Moultrie, Angeline Price, Rajendra Raman, Apirthan Rajasingam, Lauren Rothwell, Ravishankar Prabhakar Shashikala, Erica Smith, Vittoria Sorice, James van Oppen, James Wallace, Tom Young, Effie Polyzogopoulou, Lluís Llauger

**Affiliations:** 1 Division of Emergency Medicine, Department of Acute Medicine, Geneva University Hospitals, Rue Gabrielle-Perret-Gentil 4, 1211 Geneva, Switzerland; 2 Faculty of Education and Health Sciences, Ageing Research Centre, Health Research Institute, School of Allied Health, University of Limerick, Limerick, Ireland; 3 College of Life Sciences, University of Leicester, Leicester, LE1 7HA UK; 4 Belfast Health and Social Care Trust, Royal Victoria Hospital, Belfast, BT12 6BA UK; 5 Centre for Urgent and Emergency Care Research, The University of Sheffield, Sheffield, S1 4DA UK

**Keywords:** Emergency care, Frailty, Delirium, Health services

## Abstract

**Background:**

The observational Frailty in European Emergency Departments (FEED) study found 40% of older people attending for care to be living with frailty. Older people with frailty have poorer outcomes from emergency care. Current best practice calls for early identification of frailty and holistic multidisciplinary assessment. This survey of FEED sites explores variations in frailty-attuned service definitions and provision.

**Methods:**

This cross-sectional survey included study sites across Europe identified through snowball recruitment. Site co-ordinators (healthcare professionals in emergency and geriatric care) were surveyed online using Microsoft Forms. Items covered department and hospital capacity, frailty and delirium identification methods, staffing, and frailty-focused healthcare services in the ED. Descriptive statistics were reported.

**Results:**

A total of 68 sites from 17 countries participated. Emergency departments had median 30 (IQR 21–53) trolley spaces. Most defined "older people" by age 65+ (64%) or 75+ (25%). Frailty screening was used at 69% of sites and mandated at 38%. Night-time staffing was lower compared to day-time for nursing (10 [IQR 8–14] vs. 14 [IQR 10–18]) and physicians (5 [IQR 3–8] vs. 10 [IQR 7–15]). Most sites had provision for ED frailty specialist services by day, but these services were rarely available at night. Sites mostly had accessible facilities; however, hot meals were rarely available at night (18%).

**Conclusion:**

This survey demonstrated variability in case definitions, screening practices, and frailty-attuned service provision. There is no unanimous definition for older age, and while the Clinical Frailty Scale was commonly used, this was rarely mandated or captured in electronic records. Frailty services were often unavailable overnight. Appreciation of the variation in frailty service models could inform operational configuration and workforce development.

**Supplementary Information:**

The online version contains supplementary material available at 10.1186/s13049-024-01234-w.

## Introduction

The European population is ageing, and more older people are living with frailty. Frailty is present in 40% of older people (age 65+) attending European Emergency Departments (ED), varying broadly between countries from 26 to 54% [1, 2]. It is recognised that this population is often poorly served by and have poor experiences through traditional emergency care models [3, 4]. The complex nature of this cohort means they often present with undifferentiated complaints and are vulnerable to under-triage and ultimately poorer outcomes, including more frequent mortality, admissions, and longer stays [5–7]. Geriatric emergency medicine has emerged as a field of subspecialty interest with its own training curriculum, clinical guidance, and research agenda [8–10].

The core tenet of geriatric emergency medicine is a holistic approach which adopts the principles of comprehensive geriatric assessment (CGA) [11, 12]. Current, ‘traditional’, emergency care systems are not designed to deliver this at scale, typically best-serving people with single and specific injuries or illnesses rather than enabling multidisciplinary evaluation of complex and interacting problems [13, 14].

Accordingly, healthcare service models worldwide are being reconfigured to better provide for the needs of older people living with frailty. European guidelines provide advice on optimising ED care models for older people, and international accreditation programmes advocate for core processes and services [15]. However, practice and outcomes are known to vary across European EDs [16]. It is unclear to what extent these are currently adjusted to recommendations. Insight into differences in current approaches for frailty identification and service availability across European EDs could contribute to improving practice and provision.

Therefore, the aim of this project is to report on methods in use for the definition, identification, and management of frailty using a survey of European emergency departments. This project was a planned secondary objective of the FEED study, which sought primarily to evaluate the prevalence of Frailty in European Emergency Departments [2].

## Methods

### Design, recruitment and participants

This was a planned survey study performed during preparation for the FEED observational phase [17]. The FEED study recruited European emergency departments using snowball sampling (new units are recruited by other units to form part of the sample) through mailing lists (European Taskforce for Geriatric Emergency Medicine), research networks (European Geriatric Medicine Society and European Society for Emergency Medicine), and social media. A site co-ordinator at each participating department was invited to complete a survey on their service characteristics. Site co-ordinators were healthcare professionals (doctors or advanced clinical practitioners) working in emergency and geriatric care. Hospitals that did not participate in the FEED study were not included in this survey.

### Survey instrument and administration

The survey items were designed by consensus with eleven experts in geriatric emergency medicine. All were working in Europe and held current or recent leadership positions in special interest groups on acute frailty care. Items were in English and considered department and hospital capacity, frailty and delirium identification methods, typical professional staffing, and frailty-attuned healthcare services available in the ED (Supplementary Material 1). The name of the site co-ordinator was requested to minimise the risk of site duplication.

Administration of the survey was online using Microsoft Forms in the period May–June 2023. Due to the expected heterogeneity of health service models, a document of abbreviations and definitions was prepared and provided to participants (Supplementary Material 2). Three reminder emails at fortnightly intervals were sent to sites identified as potential participants, and recruited site co-ordinators were asked to complete the survey prior to collecting data for the prevalence phase of the FEED study. Service characteristics survey data were retained for those sites withdrawing from the observational phase.

### Analysis

Site characteristics were described by country, hospital and emergency department capacity (bed spaces), and the emergency department’s physician and nursing staff levels. Frailty-attuned services were described by use (recommended or mandatory) of screening tools for frailty and delirium, presence or availability of specialist professional services, and availability of departmental facilities. Summary statistics (frequencies, medians with interquartile range, and means with standard deviation as appropriate) were prepared using Stata version 17 (Stata Corp., College Station, Texas, USA) and charts using R with packages *ggplot2* and *ggmap* (R Core Team 2022). The manuscript was prepared with reference to the Consensus-Based Checklist for Reporting of Survey Studies (CROSS) (Supplemental Material 3). As this was a convenience sample, no power analysis was performed. For continuous variables, unanswered questions were considered missing and no imputation was performed. For categorical variables, responses “no” or “none” were imputed for unanswered questions.

### Regulatory approval

The study received ethical approval (University of Leicester ref 39346) and the protocol was deposited online [17]. Site co-ordinators obtained additional approvals for participation where required by local and national policies and legislation.

## Results

### Site characteristics

**Fig. 1:**
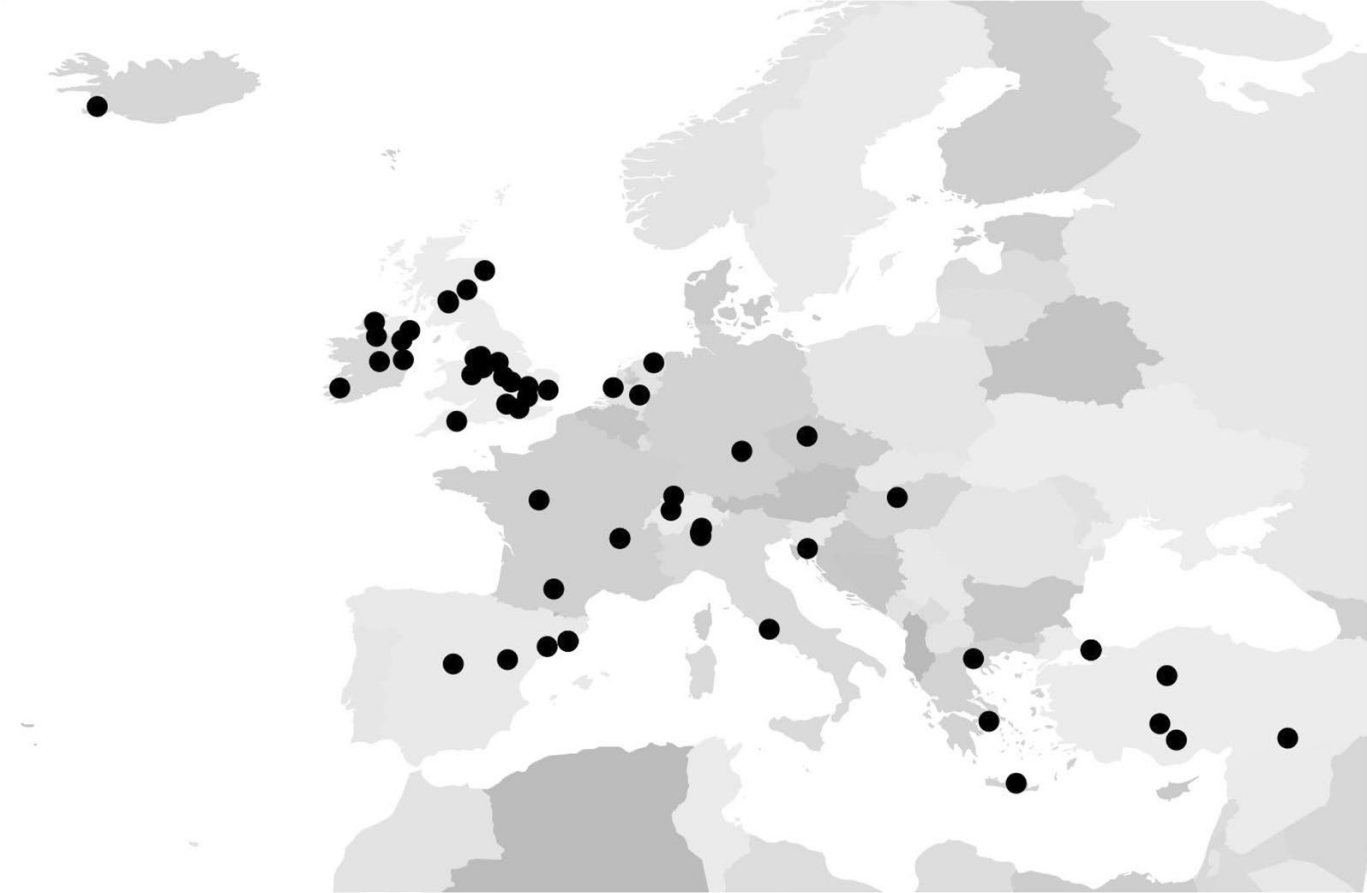
Sites distribution

Professionals representing sixty-eight sites in sixteen countries participated in the survey. Sites were spread across Europe, although North-Eastern and Scandinavian countries were not represented (Fig. 1). Departments varied widely in capacity, with a median number of 30 (IQR 21–53) trolley spaces.

Sites most commonly defined “older people" as being aged 65+ (64%) or 75+ (25%). The Clinical Frailty Scale was used at 69% of the sites, but screening for frailty was a mandatory element of care only at 38% (Table 1). Delirium screening also was rarely mandated (24%). The 4AT was the most frequently used delirium screening tool (31%). Half of the sites using electronic health records (EHR) did not have fields to capture frailty or delirium assessments.

**Table 1:** Sites characteristics

	Total (N = 68)
Country—n (%)	
UK	23 (33.8)
Spain	7 (10.3)
Turkey	7 (10.3)
Republic of Ireland	6 (8.8)
Switzerland	4 (5.9)
Belgium	3 (4.4)
France	3 (4.4)
Greece	3 (4.4)
The Netherlands	3 (4.4)
Croatia	2 (2.9)
Malta	2 (2.9)
Czech Republic	1 (1.5)
Germany	1 (1.5)
Hungary	1 (1.5)
Iceland	1 (1.5)
Italy	1 (1.5)
Number of trolleys or bed spaces in the ED—median [IQR]	30 [21–53]
Age cut-off used to define older or to screen for geriatric disease—n (%)	
60	1 (1.5)
65	43 (64.2)
67	1 (1.5)
70	4 (6.0)
75	17 (25.4)
80	1 (1.5)
Mandatory frailty screening—n (%)	
No	35 (51.5)
Yes	26 (38.2)
Partially/Unclear	7 (10.3)
Tools used to screen for frailty—n (%)	
None	14 (20.6)
Clinical Frailty Scale (CFS) only	47 (69.1)
Other tools than the CFS	6 (8.8)
Multiples tools, including the CFS	1 (1.47)
Mandatory delirium screening—n (%)	
No	52 (76.5)
Yes	16 (23.5)
Tools used to screen for delirium—n (%)	
None	20 (29.4)
4AT only	21 (30.9)
Other tools than 4AT	12 (17.6)
Multiples tools, including 4AT	15 (22.1)
ED electronical health record – n (%)	60 (88.2)
Without collection of frailty or delirium screen	31 (51.7)
With collection of delirium screen only	2 (3.3)
With collection of frailty screen only	16 (26.7)
With collection of frailty and delirium screen	11 (18.3)

The respondents’ emergency departments had one-third lower nursing staffing at night-time (10 [IQR 8–14] vs. 14 [IQR 10–18]). Meanwhile the physician staffing overnight was half that in daytime (5 [IQR 3–8] vs. 10 [IQR 7–15]) (Table 2).

**Table 2:** Staff, facilities, and resources available during day and night shifts

	Day (2PM)	Night (2AM)
ED staff		
ED nurses—median [IQR]	14 [10–18]	10 [8–14]
ED physician—median [IQR]	10 [7–15]	5 [3–8]
Professional support present or available		
Social worker	49 (72.1)	7 (10.3)
Geriatrician	43 (63.2)	7 (10.3)
Physiotherapist	43 (63.2)	6 (8.8)
Pharmacist	39 (57.4)	8 (11.8)
Geriatric nurse	33 (48.5)	2 (2.9)
Occupational therapist	32 (47.06)	0 (0.0)
Palliative care specialist	38 (55.9)	4 (5.9)
Discharge nurse	28 (41.2)	3 (4.4)
1:1 care support	20 (29.4)	15 (22.1)
ED facilities—n (%)		
Accessible toilet	64 (94.1)	64 (94.1)
Hot meal	44 (64.7)	12 (17.6)
Pressure mattress	50 (73.2)	49 (72.1)
Walking aids	53 (77.9)	44 (64.7)

### Provision of frailty-attuned services

One- to two-thirds of sites had frailty specialist services present or available to attend the ED during the day, including social workers (72%), geriatricians and geriatric specialist nurses (63%, 49%), pharmacists, physiotherapists, and occupational therapists (57%, 63%, 47%), and palliative care specialists and discharge nurses (56%, 41%) (Table 2). These services were mostly unavailable overnight (0–12% presence). The frequency of sites providing for 1:1 care support was similar at day (29%) and night (22%).

There was little diurnal variation in the availability of most department environmental facilities, with 94% having accessible toilets, 73% having pressure-relieving mattresses available, and 78% (65% overnight) able to access walking aids. Hot meals were rarely available overnight (18%).

## Discussion

This survey investigated for the first time the type of specific assessment and services for geriatric patients in European emergency departments and has demonstrated heterogeneity in case definitions, screening standards, and provision of frailty-attuned services.

Current literature in geriatric emergency medicine focuses on frailty and delirium as predictive markers for poorer outcomes from healthcare [18, 19], and yet fewer than half of sites mandated screening for these. While mandatory screening with the Clinical Frailty Score was low, this was consistent with systematic review findings around the instrument’s implementation [20]. With worldwide population ageing, presentations to emergency departments by people living with frailty will inevitably increase. While protocols and policies have been developed and implemented to improve the collaboration with other specialists, delays in people reaching these services due to resource pressures mean there remains a gap in healthcare needing to be filled by professionals competent in geriatric emergency medicine [8, 9].

Most participating departments did not have access to frailty-specialised healthcare professionals overnight, and fewer than one-fifth were able to provide hot food to a person attending at night. In the context of a worldwide crisis in emergency department crowding it is highly likely that older people living with frailty were attending and remaining in the participating departments overnight, prompting uncomfortable reflections on the likelihood of hospital-associated harms and deterioration [21].

### Limitations

The study aimed to represent Europe, and yet participation was mostly in North-Western and Southern countries. This could perhaps be due to differences in frailty prevalence and perspectives, and scope of practice and priorities for emergency care across nations. Where we corresponded with potential sites including in Scandinavia and North-Eastern Europe (suggesting the recruitment strategy reached these regions), non-participation was frequently attributed to the study’s summer timing and difficulties in obtaining local regulatory approvals.

The findings presented here may not accurately portray populations and practices in Scandinavian and North-Eastern European countries or indeed in other continents. However, this study follows national-level inquiry as the first European-level evaluation of emergency frailty care provision [22]. Further knowledge might be gained through additional international observation.

Response to surveys is often by those who have existing interest in the topic. In this case, respondents were likely to have been special interest group members or following geriatric emergency medicine themed social media accounts. Participation may therefore have been by professionals working at sites with better-established frailty practices and resources. The present study might then overestimate the true presence of frailty-attuned services and practices.

### Clinical implication

The results of this study reinforce the need for uniform practices. Despite collegiate collaboration and ambition, disparate targets, quality criteria, and data recording limit the potential for large scale comparative studies. International professional associations might therefore work towards a common core set of definitions and standards, ultimately to enable outcomes research and improvement using routine data. Implementation of established standards may improve geriatric emergency care provision through service reconfiguration and audit. While the impact on patient-reported outcomes has not yet been evaluated, the North American Geriatric Emergency Department Accreditation scheme has led to reduced admissions and healthcare costs (23, 24). These guidelines have now also been adopted in several Asian, European, and South American centres. While outcomes might be more feasibly compared using consistent core definitions and practice, the precise operationalisation currently does and inevitably will continue to vary between settings due to local demographics and available health service resources. Service configuration and innovation will require evaluation and ongoing monitoring for meaningful local effect as well as for contribution to wider scale endeavours. Emergency departments with lower provision of frailty-attuned services could refer to these results when seeking support for additional hospital resourcing.

### Research implication

On a research perspective, the issue of the impact on patients’ outcomes remains. Future studies should look at the association between frailty-attuned services and patient-reported outcomes. Researchers should also consider healthcare providers’ perspectives. While this survey was filled by each site’s representative, their vision might not be the same as others working daily in the ED (physicians, nurses, allied health professionals), especially on the importance of those services and their utilisation of frailty assessment and delirium screening results.

## Conclusion

This European survey demonstrated variability in case definitions, screening practices, and frailty-attuned service provision. There is no unanimous definition for older age. While the Clinical Frailty Scale was commonly used, this was rarely mandated or captured in electronic records. Provision of frailty-attuned specialist services was infrequent overnight. Appreciation of the variation in frailty service models could inform operational configuration and workforce development.

## Supplementary Information

Additional file 1.

## Data Availability

The data that support the findings of this study (dataset, Stata code, R code) are available from the corresponding author at reasonable request.
