# Correction: Service provision for Frailty in European Emergency Departments (FEED): a survey of operational characteristics

**DOI:** 10.1186/s13049-024-01293-z

**Published:** 2024-11-27

**Authors:** Christophe A. Fehlmann, Kara Mc Loughlin, Emma Jane Cosgrif, John Francis Ferrick, James David van Oppen, European Taskforce for Geriatric Emergency Medicine

**Affiliations:** 1 Division of Emergency Medicine, Department of Acute Medicine, Geneva University Hospitals, Rue Gabrielle-Perret-Gentil 4, Geneva, 1211 Switzerland; 2 Faculty of Education and Health Sciences, Ageing Research Centre, Health Research Institute, School of Allied Health, University of Limerick, Limerick, Ireland; 3 College of Life Sciences, University of Leicester, Leicester, LE1 7HA UK; 4 Belfast Health and Social Care Trust, Royal Victoria Hospital, Belfast, BT12 6BA UK; 5 Centre for Urgent and Emergency Care Research, The University of Sheffield, Sheffield, S1 4DA UK

**Correction to: Fehlmann et al. Scand J Trauma Resusc Emerg Med (2024) 32:64**

10.1186/s13049-024-01234-w.

Following the publication of the Original Article, the authors reported that they missed including two names from the collaborators’ list, namely **Effie Polyzogopoulou** (Greece) and **Lluís Llauger** (Spain).

The original article has been updated to include the two names on the collaborators’ list.

