# Supplementary material for: Service provision for Frailty in European Emergency Departments (FEED): a survey of operational characteristics
Source: Scand J Trauma Resusc Emerg Med. 2024 Jul 29;32:64. doi: 10.1186/s13049-024-01234-w (PMC11285136; doi:10.1186/s13049-024-01234-w)
Supplement: Supplementary file 1 — Additional file 1. [file 13049_2024_1234_MOESM1_ESM.docx]

## Supplementary Material 1: Survey items

| Hospital size and staffing | |
| --- | --- |
| 1 | Site: country |
| 2 | Site: name of the hospital |
| 3 | Site: number of inpatient beds |
| 4 | Site: number of trolley or bed spaces in the ED |
| 5 | Staff: rostered ED nurses on a typical Tuesday at 1400h |
| 6 | Staff: rostered ED nurses on a typical Tuesday at 0200h |
| 7 | Staff: rostered ED doctors on a typical Tuesday at 1400h |
| 8 | Staff: rostered ED doctors on a typical Tuesday at 0200h |
| Screening tools and health records | |
| 9 | Local age threshold for a person being “older” |
| 10 | Frailty screening tools in routine use |
| 11 | Is frailty screening mandated for older people who are in the ED? |
| 12 | The year when frailty screening tools were first used routinely |
| 13 | Delirium screening tools in routine use |
| 14 | Is delirium screening mandated for older people who are in the ED? |
| 15 | Does your ED use electronic records (for any health data)? |
| 16 | Does your ED electronic record system collect frailty and delirium screening tools? |
| Hospital and local services | |
| 17 | Select professional services which are present or available to physically attend ED at 1400h on a typical Tuesday:  1:1 care support or activities support; discharge or community liaison nurse; geriatrician; geriatric specialist nurse; occupational therapist; palliative care doctor; palliative care specialist nurse; pharmacist; physiotherapist; social worker |
| 18 | Select professional services which are present or available to physically attend ED at 0200h on a typical Tuesday:  1:1 care support or activities support; discharge or community liaison nurse; geriatrician; geriatric specialist nurse; occupational therapist; palliative care doctor; palliative care specialist nurse; pharmacist; physiotherapist; social worker |
| 19 | Select facilities available in the ED at 1400h on a typical Tuesday:  Accessible toilet; hot meal; pressure-relieving mattress; walking aids |
| 20 | Select facilities available in the ED at 0200h on a typical Tuesday:  Accessible toilet; hot meal; pressure-relieving mattress; walking aids |
| 21 | Select local (outside of hospital) services available at 1400h on a typical Tuesday:  Minor illness or injuries centre; pharmacy; general practitioner; telephone advice line |
| 22 | Select local (outside of hospital) services available at 0200h on a typical Tuesday:  Minor illness or injuries centre; pharmacy; general practitioner; telephone advice line |

## Supplementary Material 2: abbreviations and definitions

### Abbreviations

1:1 One-to-one - a healthcare worker allocated to one individual

APOP Acuut presenterende oudere patiënt (a risk screener)

CFS Clinical Frailty Scale

CI Chief Investigator

ED Emergency Department

ETGEM European Taskforce on Geriatric Emergency Medicine

GCP Good Clinical Practice

NEWS2 National Early Warning Score (v2)

OT Occupational Therapist

PT Physiotherapist

R&D Research and Development

REC Research Ethics Committee

SOP Standard Operating Procedure

Definitions

Clinical Frailty Scale

We score this for two weeks prior to the current illness / injury so that it captures the person’s baseline. Here is an excellent and quick e-learning resource: <https://rise.articulate.com/share/deb4rT02lvONbq4AfcMNRUudcd6QMts3#/>

Date and time of ED arrival

The point in time when a person physically arrives at the emergency department.

Date and time of ED departure

The point in time when a person physically leaves the emergency department. If a person remains under the care of an emergency physician but physically moves to an area dedicated for observation, intervention, or specialist review with dedicated nursing care (for example, an ED decisions unit or observation ward) then this will be considered as being admitted to hospital and is the time the person ‘leaves’ the ED.

Discharge / community liaison nurse

A qualified and/or registered healthcare professional who coordinates communication and ongoing support for the person between their care teams inside and outside of the hospital, for instance liaising with professional carers or community nurses. The professional fulfils only this role for their full shift, but may perform other roles on different shifts.

Emergency department

A healthcare service providing unscheduled assessment and treatment of acute problems which a patient or professional believes to require immediate attention. The service has facilities for resuscitation care. Typically (but not always), the service is available at any time of day or night and is overseen by a specialist physician.

Ethnic group

The ethnicity category with which the person identifies. The categories are from the UK Office for National Statistics. A category ‘Other’ is available.

Geriatric specialist nurse

A qualified and/or registered healthcare professional with postgraduate experience, training, or qualification in assessment and intervention for older people. The professional assesses and treats only older people for their full shift, but may perform other roles on different shifts. Include here professionals from non-nursing backgrounds who perform this advanced role, for example physiotherapists who work as Advanced Clinical Practitioners.

Minor illness or injuries centre

A healthcare service providing unscheduled assessment and treatment of acute problems which a patient or professional believes to be non-life threatening. Typically, the service is not provided by specialist physician and is instead delivered by autonomous nurse specialists or general practitioners. These are located and staffed separately from emergency departments, although a hospital may provide both an emergency department and a minor illness centre.

Mode of attendance

The mechanism by which a person arrives at the emergency department, including self-presentation, arrival by ambulance, or referral from a healthcare professional (for example, a general practitioner).

Professional care at home

The person receives care where they live, in any form (for example, dressing, cleaning, or shopping) from a person who is paid a salary by either the person themselves or through state-funded support. This is different from the ‘informal’ care given by family, friends, or neighbours for which a salary is not paid.

Residential care facility

A place of residence staffed with professional carers day and night. People may live here permanently or temporarily (for example, for rehabilitation following an injury). Carers may or may not be qualified and/or registered. Other terms include ‘assisted living facility’, ‘care home’, ‘nursing home’, and ‘rest home’.

Resuscitation room at any point

A yes or no response for whether the person received any care in an area of the emergency department dedicated for resuscitation. For yes, the person may or may not have received ‘critical care’, but was physically located in the resuscitation room and assessed and/or treated by nursing or medical staff in that area.

Rostered ED doctors

Qualified doctors of any grade, who are responsible for seeing new patients in the emergency department.

Example: overnight there are 0 consultants or specialists, 4 residents or specialty trainees, and 6 foundation or general practice trainees seeing new arrivals to the ED. We report 10 rostered ED doctors at 2am.

Rostered ED nurses

Qualified and/or registered nurses responsible for providing care to patients in the emergency department. Unqualified workers are not included, for example healthcare assistants and care support workers.

Telephone advice line

A healthcare service which patients can consult for triage, assessment, referral, or self-management advice of problems which they consider not to be life-threatening. This is not the 112/999 service through which patients call for an ambulance, but the outcome of the advice line may include dispatch of an ambulance. This service may be provided by healthcare professionals or by trained staff following algorithms.

## Supplementary Material 3: CROSS Checklist

| **Section/topic** | **Item** | **Item description** | **Reported on page #** |
| --- | --- | --- | --- |
| **Title and abstract** | | |  |
| Title and abstract | 1a | State the word “survey” along with a commonly used term in title or abstract to introduce the study’s design. | 1 |
|  | 1b | Provide an informative summary in the abstract, covering background, objectives, methods, findings/results, interpretation/discussion, and conclusions. | 2 |
| **Introduction** | | |  |
| Background | 2 | Provide a background about the rationale of study, what has been previously done, and why this survey is needed. | 3 |
| Purpose/aim | 3 | Identify specific purposes, aims, goals, or objectives of the study. | 3 |
| **Methods** | | |  |
| Study design | 4 | Specify the study design in the methods section with a commonly used term (e.g., cross-sectional or longitudinal). | 4 |
|  | 5a | Describe the questionnaire (e.g., number of sections, number of questions, number and names of instruments used). | 4 |
| Data collection methods | 5b | Describe all questionnaire instruments that were used in the survey to measure particular concepts. Report target population, reported validity and reliability information, scoring/classification procedure, and reference links (if any). | 4 |
|  | 5c | Provide information on pretesting of the questionnaire, if performed (in the article or in an online supplement). Report the method of pretesting, number of times questionnaire was pre-tested, number and demographics of participants used for pretesting, and the level of similarity of demographics between pre-testing participants and sample population. | Not reported. |
|  | 5d | Questionnaire if possible, should be fully provided (in the article, or as appendices or as an online supplement). | 15 |
| Sample characteristics | 6a | Describe the study population (i.e., background, locations, eligibility criteria for participant inclusion in survey, exclusion criteria). | 4 |
|  | 6b | Describe the sampling techniques used (e.g., single stage or multistage sampling, simple random sampling, stratified sampling, cluster sampling, convenience sampling). Specify the locations of sample participants whenever clustered sampling was applied. | 4 |
|  | 6c | Provide information on sample size, along with details of sample size calculation. | 5 |
|  | 6d | Describe how representative the sample is of the study population (or target population if possible), particularly for population-based surveys. | NA |
| Survey  administration | 7a | Provide information on modes of questionnaire administration, including the type and number of contacts, the location where the survey was conducted (e.g., outpatient room or by use of online tools, such as SurveyMonkey). | 5 |
|  | 7b | Provide information of survey’s time frame, such as periods of recruitment, exposure, and follow-up days. | 5 |
|  | 7c | Provide information on the entry process:  –>For non-web-based surveys, provide approaches to minimize human error in data entry.  –>For web-based surveys, provide approaches to prevent “multiple participation” of participants. | 4 |
| Study preparation | 8 | Describe any preparation process before conducting the survey (e.g., interviewers’ training process, advertising the survey). | 4 |
| Ethical considerations | 9a | Provide information on ethical approval for the survey if obtained, including informed consent, institutional review board [IRB] approval, Helsinki declaration, and good clinical practice [GCP] declaration (as appropriate). | 5 |
|  | 9b | Provide information about survey anonymity and confidentiality and describe what mechanisms were used to protect unauthorized access. |  |
| Statistical  analysis | 10a | Describe statistical methods and analytical approach. Report the statistical software that was used for data analysis. | 4-5 |
|  | 10b | Report any modification of variables used in the analysis, along with reference (if available). |  |
|  | 10c | Report details about how missing data was handled. Include rate of missing items, missing data mechanism (i.e., missing completely at random [MCAR], missing at random [MAR] or missing not at random [MNAR]) and methods used to deal with missing data (e.g., multiple imputation). | 6 |
|  | 10d | State how non-response error was addressed. |  |
|  | 10e | For longitudinal surveys, state how loss to follow-up was addressed. | NA |
|  | 10f | Indicate whether any methods such as weighting of items or propensity scores have been used to adjust for non-representativeness of the sample. | NA |
|  | 10g | Describe any sensitivity analysis conducted. | NA |
| **Results** | | |  |
| Respondent characteristics | 11a | Report numbers of individuals at each stage of the study. Consider using a flow diagram, if possible. | NA |
|  | 11b | Provide reasons for non-participation at each stage, if possible. | NA |
|  | 11c | Report response rate, present the definition of response rate or the formula used to calculate response rate. | NA |
|  | 11d | Provide information to define how unique visitors are determined. Report number of unique visitors along with relevant proportions (e.g., view proportion, participation proportion, completion proportion). | NA |
| Descriptive  results | 12 | Provide characteristics of study participants, as well as information on potential confounders and assessed outcomes. | 6 |
| Main findings | 13a | Give unadjusted estimates and, if applicable, confounder-adjusted estimates along with 95% confidence intervals and p-values. | NA |
|  | 13b | For multivariable analysis, provide information on the model building process, model fit statistics, and model assumptions (as appropriate). | NA |
|  | 13c | Provide details about any sensitivity analysis performed. If there are considerable amount of missing data, report sensitivity analyses comparing the results of complete cases with that of the imputed dataset (if possible). | NA |
| **Discussion** | | |  |
| Limitations | 14 | Discuss the limitations of the study, considering sources of potential biases and imprecisions, such as non-representativeness of sample, study design, important uncontrolled confounders. | 7 |
| Interpretations | 15 | Give a cautious overall interpretation of results, based on potential biases and imprecisions and suggest areas for future research. | 8 |
| Generalizability | 16 | Discuss the external validity of the results. | 8 |
| **Other sections** | | |  |
| Role of funding source | 17 | State whether any funding organization has had any roles in the survey’s design, implementation, and analysis. | 15 |
| Conflict of interest | 18 | Declare any potential conflict of interest. | 15 |
| Acknowledgements | 19 | Provide names of organizations/persons that are acknowledged along with their contribution to the research. | 15 |
